# Barriers to and facilitators of women GPs’ careers: a systematic review

**DOI:** 10.3399/BJGPO.2024.0282

**Published:** 2025-07-02

**Authors:** Laura Jefferson, Elin Webster, Su Golder, Katie Barnett, Nicola Greenwood, Veronica Dale, Karen Bloor

**Affiliations:** 1 Department of Health Sciences, University of York, England, UK; 2 Academic Foundation Doctor, York Teaching Hospital, England, UK; 3 Haxby Group, England, UK

**Keywords:** general practice, systematic review, primary healthcare, gender

## Abstract

**Background:**

Despite women comprising 52% of full-time equivalent GPs in England, a significant gender pay gap persists (15% after adjustments). Further understanding of the barriers and facilitators impacting women GPs’ careers is needed.

**Aim:**

To identify and synthesise research evidence exploring barriers to and facilitators of women GPs’ careers.

**Design & setting:**

Systematic review of qualitative and quantitative studies. Studies were included of GPs conducted in the UK NHS general practice setting.

**Method:**

Review methods followed Cochrane and Preferred Reporting Items for Systematic reviews and Meta-Analyses (PRISMA) guidelines to systematically search MEDLINE, Embase, Health Management Information Consortium (HMIC), and Google Scholar to identify studies that explored gendered barriers and facilitators to GP careers. An inductive thematic analysis was used to synthesise the evidence.

**Results:**

Twenty-one articles were included in this review, with varied study designs. No relevant intervention studies were identified. There was a lack of recent research evidence; over half of the studies were conducted more than 20 years ago. Most met quality criteria, although there were some problems with reporting and adjustment for potential confounders. Studies found barriers at personal, socio-cultural, and system levels that inhibit women GPs’ careers. While some positive changes have been documented across studies that span some 30 years, many challenges remain.

**Conclusion:**

Despite general practice being a medical specialty where women outnumber men, barriers at personal, socio-cultural, and system levels continue to inhibit women GPs’ careers.

## How this fits in

Women now constitute the majority of full-time equivalent GPs in England, yet a substantial gender pay gap persists, highlighting potentially persisting inequalities within the profession. Existing research has predominantly focused on hospital specialties, with limited exploration of barriers specific to women in general practice. This study synthesises UK-based evidence to identify factors influencing women GPs’ careers, including societal expectations, workplace cultures, and the differential uptake of partner roles. By exploring these evidence gaps, the findings provide critical insights to inform policies aimed at promoting gender equity in general practice and reducing the gender pay gap.

## Introduction

Women constitute 52% of full-time equivalent GPs in England^1^ and yet a substantial gender pay gap of 33.5% (unadjusted) exists, which is one of the highest of any UK profession.^2^ While this largely highlights differences in working hours, age and experience, a 15% adjusted pay gap remains. The presence of a ‘glass ceiling’ in medicine has been widely described, referring to women doctors’ apparent constrained ability to progress in their careers, and worse reported pay and conditions.^3–7^ In the general practice setting, we know that women are less likely to take on partner (formally termed ‘principal’) roles that are associated with higher pay profit-sharing.^1,2^ Studies exploring gender differences in medical careers have tended, though, to focus on hospital specialties, particularly those with historically lower proportions of women. In surgical specialties, for example, studies have found that discrimination against women doctors remains, with apparent differential treatment and ‘old boys’ clubs’.^8–10^ Hafferty^11^ described a ‘hidden curriculum’ of cultural norms and customs in medical institutions some 25 years ago, but a recent British Medical Association (BMA) report on sexism in medicine highlights a worryingly persistent negative culture in today’s medical system: 91% of women doctors reported experiencing sexism at work.^12^

The impact of wider societal gender expectations creates differential tensions for women doctors, particularly in relation to caring responsibilities, even in dual doctor marriages.^13^ Evidence from international primary care settings recently suggested that this societal expectation places additional pressure on women GPs at life transitions.^14^ In the UK, recent research is lacking on this topic, and the wide gender pay gap in general practice^2^ highlights a need to explore the barriers and facilitators that influence women GPs’ careers. As part of a wider UK policy research project looking at differential uptake of GP partner roles by women GPs,^15^ we undertook a systematic review of the existing UK evidence to identify evidence gaps and provide a synthesis of the key barriers to and facilitators of women GPs’ careers.

## Method

We used systematic review methods, following the Cochrane guidelines for conducting systematic reviews^16^ and, to ensure transparency of reporting, we used a Preferred Reporting Items for Systematic reviews and Meta-Analyses (PRISMA) checklist.^17^ To reduce potential duplication of effort, we registered the study in advance (PROSPERO CRD42023384176).

### Search strategy

We employed a varied search strategy, using both database searching and wider sources to search for reports. Our sources included MEDLINE, Embase, and the Healthcare Management Information Consortium (HMIC) database (initial search 5 January 2022, repeated 4 January 2023), alongside searches of Google Scholar, key websites, reference lists, and online e-theses (via EThOs) to capture grey literature. See Supplementary Table S2 for full search strategies. We conducted forward and backward citation searching on included studies. No date or language restrictions were applied.

### Inclusion criteria

We included studies if they investigated barriers to and facilitators of women GPs’ careers, including, but not limited to, uptake of partnership roles. All GP types were included, we did not exclude trainees or focus by contract type. We included studies that either explore specifically the experiences of women or draw comparisons between genders. We excluded studies of multiple health professional groups if GP findings were not disaggregated. Eligible studies were focused on those conducted in UK general practice or primary care settings, with non-UK studies excluded owing to significant differences in how healthcare systems are delivered internationally. No limits were applied according to study design, but we included only empirical research evidence, excluding case reports and editorials.

### Selection of studies

We downloaded search results into Covidence^18^ to de-duplicate and conduct screening. Two of five reviewers independently completed initial screening of titles and abstracts, followed by full-text screening. We resolved any disagreements between reviewers through discussion or a third reviewer (LJ or SG).

### Data extraction and quality assessment

We used a pre-piloted data extraction form, with one of four reviewers extracting data and cross-checking a 20% sample to ensure consistency. Depending on the study design, we used the Joanna Briggs Institute (JBI) Checklist for Analytical Cross-Sectional Studies^19^ or the Critical Appraisal Skills Programme (CASP) checklist tool for qualitative studies^20^ for quality assessment. Two reviewers independently performed quality appraisal, with arbitration by a third reviewer in cases of disagreement (5%). Studies were not excluded based on quality.

### Data synthesis

To summarise the study findings we used narrative synthesis, as variation across studies prohibited the use of quantitative approaches. We managed and sorted data in MS Excel and then employed thematic qualitative synthesis to analyse findings.

We used an iterative process, moving through the stages of initial ‘free coding’ to more descriptive and then later, analytical themes. Each stage was undertaken with regular consultation and discussion between researchers who had methodological and topic expertise, some of whom are female doctors.

## Results

### Search results

**Figure 1.**
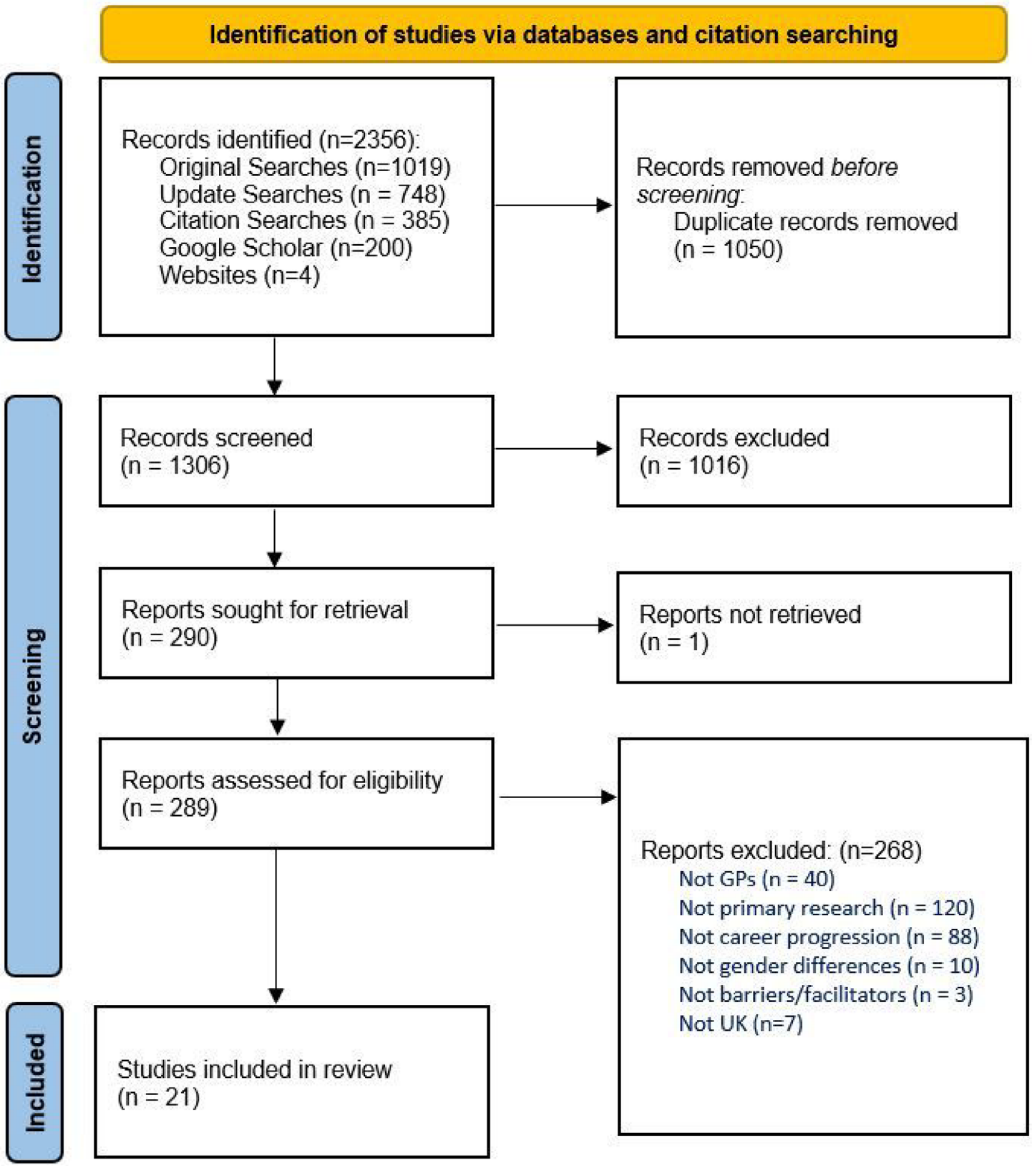
Preferred Reporting Items for Systematic reviews and Meta-Analyses (PRISMA) diagram for this study’s search and selection process

In total, we identified 2356 studies from databases and grey literature searching. After removal of duplicates, 1306 articles were screened as titles and abstracts. We excluded 1017 at this initial stage, leaving 289 for full-text review. Twenty-one studies met the inclusion criteria and were included in this review (Figure 1, Supplementary Table S1).^12,15,21–39^

### Study characteristics

Study designs varied, with 10 cross-sectional surveys, two secondary econometric analyses, one discrete choice experiment, five qualitative interview studies, and three mixed-methods studies. We found no relevant intervention studies.

Most of the studies were conducted some time ago; more than half were over 20 years ago and only three studies were conducted in the past 10 years.^12,15,29^ Of these, one was a PhD thesis that only included four GPs.^29^ Studies were geographically dispersed across the UK, with five UK-wide, four in England, one England and Wales, three in Scotland, and eight in single locations within the UK.

Six studies included only women, while the remaining 15 studies explored gender differences. Sample sizes ranged from a qualitative study with four GPs to an econometric analysis of 2271 GPs (median 368).

### Quality assessment

The quality of studies was generally good, with all providing valuable insights (Tables 1 and 2). Although all but one cross-sectional study identified potential confounding factors, only *n* = 6/13 used strategies for dealing with such confounders, for example, through statistical analyses. All other components of the quality assessment of cross-sectional studies were generally good. Qualitative studies were all judged to be valuable, and most were rigorously conducted. One study conducted in 1989 was rated as ‘unclear’ or inadequate across numerous categories^36^ and insufficient detail about analysis hindered our ability to assess trustworthiness of findings in three studies.^27,36,39^ Qualitative studies tended not to consider relationships between researchers and participants reflexively, and only three described ethical considerations.^15,29,33^

**Table 1.** Quality appraisal of quantitative studies

Author (year)	Were the criteria for inclusion in the sample clearly defined?	Were the study subjects and the setting described in detail?	Was the exposure measured in a valid and reliable way?	Were objective, standard criteria used for measurement of the condition?	Were confounding factors identified?	Were strategies to deal with confounding factors stated?	Were the outcomes measured in a valid and reliable way?	Was appropriate statistical analysis used?
Baker *et al* (1995)^24^	Yes	Yes	Yes	Yes	Yes	No	Yes	Yes
BMA (2021)^12^	Yes	Unclear	Yes	Yes	Yes	Yes	Unclear	Yes
Brown *et al* (1983)^31^	Yes	Unclear	Yes	Yes	Yes	No	Yes	No
French *et al* (2005)^32^	Yes	Yes	Yes	Yes	Yes	No	Yes	Yes
French *et al* (2006)^22^	Yes	Yes	Yes	Yes	Yes	Yes	Yes	Yes
Gravelle *et al* (2011)^23^	Yes	Yes	Yes	Yes	Yes	Yes	Yes	Yes
Johnson *et al* (1993)^26^	Yes	Yes	Yes	Yes	Yes	No	Yes	Yes
Johnson *et al* (1998)^25^	Yes	Yes	Yes	Yes	No	No	Yes	Yes
Leese *et al* (2002)^34^	Yes	Yes	Yes	Yes	Yes	Yes	Yes	Yes
Morris *et al* (2011)^37^	Yes	Yes	Yes	Yes	Yes	Yes	Yes	Yes
Osler (1991)^28^	Yes	Yes	Yes	Yes	Yes	No	Yes	Yes
Wedderburn *et al* (2013)^30^	Yes	Yes	Yes	Yes	Yes	No	Yes	Yes
Wordsworth *et al* (2004)^38^	Yes	Yes	Yes	Yes	Yes	Yes	Yes	Yes

BMA = British Medical Association

**Table 2.** Quality appraisal of qualitative studies

Author (year)	Section A: Are the results valid?						Section B: What are the results?			Section C: Will the results help locally?
	Was there a clear statement of the aims of the research?	Is a qualitative methodology appropriate?	Was the research design appropriate to address the aims of the research?	Was the recruitment strategy appropriate to the aims of the research?	Was the data collected in a way that addressed the research issue?	Has the relationship between researcher and participants been adequately considered?	Have ethical issues been taken into consideration?	Was the data analysis sufficiently rigorous?	Is there a clear statement of findings?	How valuable is the research?
Brooks (1998)^21^	Yes	Yes	Yes	Yes	Yes	Unclear	Unclear	Yes	Yes	Valuable
Jefferson *et al* (2022)^15^	Yes	Yes	Yes	Yes	Yes	Yes	Yes	Yes	Yes	Valuable
Lawrence (1987)^33^	Yes	Yes	Yes	Yes	Yes	Yes	Yes	Yes	Yes	Valuable
Newman (2011)^27^	Yes	Yes	Yes	Unclear	Yes	Unclear	Unclear	Unclear	Yes	Valuable
Pinder (1998)^^35^^	Yes	Yes	Yes	Yes	Yes	Unclear	Unclear	Yes	Yes	Valuable
Warren and Wakeford (1989)^36^	No	Yes	No	Unclear	Unclear	Unclear	Unclear	Unclear	Yes	Valuable
Watts (2018)^29^	Yes	Yes	Yes	Yes	Yes	Yes	Yes	Yes	Yes	Valuable
Young *et al* (2001)^39^	Yes	Yes	Yes	Yes	Yes	Unclear	Unclear	Unclear	Yes	Valuable

### Narrative synthesis

#### Personal circumstances

Studies historically focused on individuals’ personal circumstances that were described as influencing women’s choices in their careers, which were primarily the challenges associated with balancing family and work lives, but also financial barriers. Sixteen of the included studies outlined issues relating to the tendency for women GPs to bear greater family responsibilities, citing these commitments as a reason for not pursuing roles as partner (principal) in a practice, with difficulties establishing work–family balance and challenges of working full-time.^12,15,22,24–26,28–31,33–36,38,39^ Osler^30^ found GP trainees were more likely to leave training posts owing to personal challenges such as childcare and geographical demands of moving with spouse or partner’s role.

Attitudes were perceived as shifting,^15,29,33,39^ although recent research shows that gendered barriers are still strongly associated with caring responsibilities.^15^ Longitudinal cohorts reported lowering impact of childcare responsibilities on women doctors’ careers over their life course,^25,26^ but almost half of women aged >50 years still reported childcare challenges^30^ and caring responsibilities for adult dependants.^39^

Financial barriers were raised as an issue by women GPs in seven studies,^15,23,29,32,33,36,37^ often focusing on their lower earnings compared with men but also culturally gendered barriers including reluctance to negotiate pay. Statistically and economically significant lower incomes for women GPs were reported and were largely unexplained by observable characteristics.^23,32,37^ Further description, and possible reasons for lower earnings cited by studies, are described in Table 3.

**Table 3.** Further detailed findings

Personal circumstances	Reasons for **differential earnings** included women being less likely to negotiate pay,^15^ women experiencing financial exploitation in group practices with male senior partners,^33^ women being less motivated by financial gain,^15,33^ and lack of financial security in partnerships when taking maternity leave.^15,29,36^ At a personal level, these challenges related to the personal cost of taking time off for maternity leave, but also paying locum cover for GP partners.^15,29,36^ Jefferson *et al* ^15^ described women’s lower comparative earnings owing to maternity leave and part-time work resulting in reduced financial incentive to progress careers in comparison with spouses, particularly those in dual-doctor marriages. Knowledge of the New to Partnership Payment Scheme to encourage uptake of partnership roles through a financial incentive of £20 000 to new partners has been described as insufficient amidst wider uncertainty within the profession.^15^ **Childcare costs** were a barrier in two studies. Young *et a*l^39^ found salaried contracts were preferable owing to fixed incomes simplifying the financial planning for childcare costs.^39^ Leese *et al* ^34^ suggest that improved childcare and term-time contracts may encourage re-entry to partnerships. Six studies cited **spousal job location** as a barrier to progression. Gender differences were seen in the influence of a spouse’s work location determining job role;^31^ the likelihood of leaving a partnership role;^34^ and in reducing career progression and aspirations.^22,25,32^
Systemic barriers, cultures, and discrimination	Several studies described specific challenges around taking **maternity leave**. Women GPs exited training pathways owing to perceived ‘unwritten rules’ around maternity leave during training,^28,36^ historical contractual arrangements, which restricted entitlement to maternity leave^36^ and women reported a lack of support or outright negativity from colleagues when broaching maternity leave.^12,35^ Financial insecurity associated with maternity leave was highlighted across studies despite large time lags and, presumably, cultural shifts in the workplace.^15,29,36^ Historically, one study (from 1987) described challenges of out-of-hours working in obtaining childcare at short notice, with reliance on spouse or wider family during these times.^33^ Wordsworth *et al*’s discrete choice experiment (DCE),^38^ which asked salaried and partnered GPs about their preferences for hypothetical job components, found **flexibility of hours** associated with salaried roles to be a priority in career choices. Similar findings were reported in a pilot DCE by Jefferson *et al*,^15^ with both men and women GPs indicating a preference for flexible working to promote partnership uptake. In studies comparing men and women, inflexible work hours were perceived as a greater barrier to progression by women, with significant gender differences recorded in three studies.^25,26,34^
Satisfaction in role	Osler reported that more women than men (39% versus 24%) were working in a role that was not their original choice of work.^28^ Jefferson *et al* ^15^ found women GPs held more responsibility for supporting teams; further increasing workload. Johnson *et al* ^26^ reported other potential reasons for dissatisfaction, including longer time to become a GP principal for women compared with men and frustration related to the socio-cultural barriers women face in the workplace.

#### Systemic barriers, cultures, and discrimination

Socio-cultural and systemic barriers to women’s careers were found across studies. These relate to maternity leave practices, including ‘unwritten rules’ and contractual challenges,^12,28,35,36^ spousal job location,^15,22,25,31,32,34^ childcare costs,^15,24^ flexibility of roles,^15,24–26,33,34,38^ cultural challenges within general practice, and also overt discrimination^12,15,21,26,27,33,35,36^ (Table 3).

Ten studies discussed flexibility in working hours as a barrier to women GPs’ careers.^15,24–27,29,30,33,34,38^ Prior to 2004, GP partners were personally responsible for providing or organising a 24/7 service for patients.^40^ Several studies conducted pre-2004 cited out-of-hours working as a barrier to taking on a role as a GP partner,^24,34,38^ owing to the challenges of securing childcare. Flexibility in hours was a priority for GPs in reported discrete choice experiments about preferences for career choices,^15,38^ with lower out-of-hours work being a greater priority for both sessional (salaried) and principal (partner) women GPs, compared with men.^38^ While earlier studies found women GPs were statistically significantly more likely to report inflexible hours as a career barrier than men;^25,26,34^ the most recent study to explore flexibility found that although still higher among women, gender differences in preferences for flexibility in working hours was marginal, indicating a potential shift in appetite over time.^25^

Discriminatory cultures included the following: negative views of part-time working;^27,35^ increasing demarcations between salaried and partner GPs;^15^ stereotypical gendered roles in practices;^15^ and societal expectations of a doctor being male.^21,33^ Studies gave accounts of this being displayed through women’s voices not feeling heard,^15,27^ passive lack of support,^27^ differential treatment and respect from support staff,^12,15,33^ reduced opportunities for leadership roles,^12,27^ discriminatory interview practices,^12,36^ and historical marginalisation and exclusionary behaviours.^21,26,27^ Only 20% of GPs reported never experiencing sexism.^12^

There was limited exploration of intersectionality, for example, considering gender and ethnicity together. In one study, Asian women GPs cited cultural expectations about responsibilities in the home, and for Asian men, greater financial pressures around working hours owing to socialised gender roles.^25^ While the BMA report *Sexism in Medicine* found higher reports of sexism as a barrier to career progression in Black and Asian doctors (40% and 50%, compared with 23% in White doctors), this was not disaggregated by gender.^12^

#### ‘Women’s work’

Although described to a lesser extent, some studies touched on feelings of frustration that had led to dissatisfaction among women GPs. These included frustration with being given a higher burden of ‘women’s work’; particularly caseloads relating to women’s, children’s, and mental health as a result of normative assumptions.^12,15,21,26,33^ This was viewed as increasing their workload and involving longer appointment times,^15,33^ and was associated with lower professional status^21^ and overall, decreased satisfaction.^12^

#### Facilitators of women’s careers

Although reported to a lesser degree to barriers, five studies also described facilitators of women GPs’ careers.^15,27,29,30,34^ Two studies highlighted the importance of strong role models to promote positive workplace cultures, inspiring and supporting women in the workplace.^15,27^ Newman^27^ particularly stresses the need for leadership development early in GP careers, for example, through fellowship positions to encourage equal opportunities for male and female doctors to enter leadership roles. Studies also described the potential benefits of flexible working practices that encouraged re-entry to principal posts^34^ after temporary exit, for example, through ‘ramp-on and ramp-off schemes’,^27^ which may also encourage later retirement.^30^ Meanwhile, part-time or salaried roles were described as increasingly being used to cope with challenging working lives and to reduce burnout.^15,29^ Wedderburn *et al* ^30^ found greater social support (for example, from a home-based husband, or proximity of extended family) was described as ‘buffering’ childcare demands and workplace stress.

## Discussion

### Summary

This review highlights barriers at personal, socio-cultural, and system levels that inhibit women GPs’ careers. While some positive changes have been documented across studies that span some 30 years, many challenges remain. Most frequently these relate to historically gendered roles in the home and, depending on age and situation, the associated challenges of childcare responsibilities and flexible working. Wider barriers owing to medical cultures also appear slow to change; accounts of discriminatory and prejudiced behaviours are still alarmingly common.^12,15,41^ Financial constraints were described, in terms of women’s lower comparative earnings, financial pressures associated with maternity leave, and women’s lower willingness to negotiate pay.

No evaluations of interventions to support women GPs’ careers were identified in this review and there was a general lack of recent evidence that needs to be addressed. This is particularly important given the ongoing issues of GP wellbeing and retention, with evidence highlighting a differential impact on women GPs’ wellbeing across international studies.^42^

### Strengths and limitations

A key strength of this review is the systematic approaches utilised throughout searching, data extraction, and synthesis. Although our research focused on the experiences of UK doctors in general practice, findings relating to gendered medical cultures, childcare roles, and pay negotiation are likely to translate to wider settings, both in family practice internationally and wider medical cultures. Some elements may be unique to the UK context, for example, barriers to partner or principal roles.

While all contributing authors were women, we engaged academic and medical doctors, which aided our interpretation of findings.

Our review found a particular lack of recent quantitative data analysis exploring women GPs’ experiences in their careers. Further research is now needed that takes account of GP characteristics such as sex and gender, underexplored in the cross-sectional studies we identified, indeed six did not even include men as a comparison group. Recent focus on integrating sex and gender in the design of research, initiated by UK research funders, is welcome to ensure research addresses equality and inclusion.^43^

### Comparison with existing literature

To our knowledge, this is the first systematic review of UK literature on this topic. While there were few recent studies identified in this setting, some similarities across findings from recent studies highlight the ongoing social and cultural challenges that women doctors face in medical workplaces, replicating those from specialties with historically lower proportions of women doctors.^10,44^

A key area of policy focus is needed around flexible working and job crafting in general practice to support women GPs’ careers. While national workforce data suggests declining numbers of ‘full-time’ GPs over the past decade, recent analysis of GP working hours shows trends of increased working hours per ‘session’ and women GPs are working longer hours per session.^45^ Defined by the BMA as 4 hours 10 minutes,^46^ in reality mean session length is 6 hours 12 minutes.^45^ Gender differences in caseload and wider organisational roles seen as ‘women’s work’ described in this review warrant exploration at practice levels. Managers could explore variations in case-mix and task functions through internal audits to ensure equality in workload distribution. Further, approaches to job crafting that align employees’ personal and career preferences could improve satisfaction and retention, as outlined in a recent report on job crafting and flexible working in general practice.^47^

### Implications for research and practice

This review suggests that general practice workplaces should consider approaches to foster environments to support women GPs’ careers. While no intervention studies exist at present, we describe key practical solutions that may promote greater equality and inclusion in the context of general practice.

Empirical evaluation of all such schemes is required. Further in-depth qualitative work is also needed to understand the mechanisms that may support women GPs, as limited research has explored facilitators and examples of good practice.

Practices as employers should develop psychologically safe environments where women feel comfortable discussing and negotiating pay with colleagues, with greater standardisation of partner contracts that offer financial security during periods of maternity leave. While the New to Partnership Payment Scheme was introduced in 2020 to provide financial incentive and training to support greater uptake of partnership roles in general practice,^48^ knowledge of this scheme remains low. In addition, the financial incentive of £20 000 to new partners has been described as insufficient amidst wider uncertainty within the profession.^15^

Portfolio roles may offer an opportunity for greater diversity in GPs’ roles and higher earnings,^41^ and reduced intentions to leave practice.^49^ Nevertheless, Kelly *et al* ^50^ urge caution around over-focusing ‘special interest’ portfolio careers that risk losing expert generalist skills. Further work is needed to explore gender breakdown in these roles and their wider implications for GP careers and the future workforce pipeline.

Our review highlights a need to support women GPs during difficult transition periods of their careers and offer opportunities for role modelling to reduce socio-cultural barriers to career progression. It is therefore concerning that the New to Practice Fellowship scheme has recently ended,^51^ particularly since evidence highlights particular challenges facing this cohort, including low conversion rates of GP trainers to GP joiners in England.^52^ Other schemes, including the GP Supporting Mentors Scheme and New to Partnership Payment Scheme continue, although accessing such schemes requires awareness of these opportunities, time, and supportive organisational leadership.

In conclusion, despite general practice being a medical specialty where women outnumber men, barriers at personal, socio-cultural, and system levels continue to inhibit women GPs’ careers. Given the wider retention issues facing general practice and the large proportion of women in this sector, it is essential that organisational policies adapt to support this workforce. Potential opportunities exist through job crafting, flexible working, mentoring, and fellowships, but there needs to be support and leadership to encourage these opportunities. Empirical research evaluating such approaches and offering examples of good practice may provide an opportunity to strengthen this debate.

## Data

Data used for this review are available on request.

## Review registration

PROSPERO ID: CRD42023384176

## References

[1] NHS England (2023). General practice workforce. https://digital.nhs.uk/data-and-information/publications/statistical/general-and-personal-medical-services.

[2] Dacre J, Woodhams C, Atkinson C (2020). Mend the gap: the independent review into gender pay gaps in medicine in England. https://assets.publishing.service.gov.uk/media/5fd893a7e90e076631fb2286/Gender_pay_gap_in_medicine_review.pdf.

[3] Kvaerner KJ, Aasland OG, Botten GS (1999). Female medical leadership: cross sectional study. BMJ.

[4] British Medical Association (BMA) (2004). Women in academic medicine: challenges and issues.

[5] Levinson W, Lurie N (2004). When most doctors are women: what lies ahead?. Ann Intern Med.

[6] Carnes M, Morrissey C, Geller SE (2008). Women’s health and women’s leadership in academic medicine: hitting the same glass ceiling?. J Womens Health (Larchmt).

[7] Edmunds LD, Ovseiko PV, Shepperd S (2016). Why do women choose or reject careers in academic medicine? A narrative review of empirical evidence. Lancet.

[8] Davies K (2003). The body and doing gender: the relations between doctors and nurses in hospital work. Sociol Health Illn.

[9] Cassell C (1998). The woman in the surgeon’s body.

[10] Jefferson L, Bloor K, Spilsbury K (2015). Exploring gender differences in the working lives of UK hospital consultants. J R Soc Med.

[11] Hafferty FW (1998). Beyond curriculum reform: confronting medicine’s hidden curriculum. Acad Med.

[12] British Medical Association (BMA) (2021). Sexism in medicine.

[13] Soares A, Thakker P, Deych E (2021). The impact of COVID-19 on dual-physician couples: a disproportionate burden on women physicians. J Womens Health (Larchmt).

[14] Shiner A, Watson J, Doohan N, Howe A (2020). Learning or leaving? An international qualitative study of factors affecting the resilience of female family doctors. BJGP Open.

[15] Jefferson L, Golder S, Sivey P (2022). Exploring gender differences in uptake of GP partnership roles. https://www.york.ac.uk/media/healthsciences/images/research/prepare/reportsandtheircoverimages/Exploring%20gender%20differences%20in%20%20uptake%20of%20GP%20partnership%20roles.pdf.

[16] Higgins J, Thomas J, Chandler J (2021). Cochrane handbook for systematic reviews of interventions (version 6.2). https://training.cochrane.org/handbook.

[17] Moher D, Liberati A, Tetzlaff J (2009). Preferred reporting items for systematic reviews and meta-analyses: the PRISMA statement. BMJ.

[18] Covidence systematic review software VHI, Melbourne, Australia. https://www.covidence.org/.

[19] Joanna Briggs Institute (2017). Joanna Briggs Institute Checklist for Analytical Cross Sectional Studies. https://jbi.global/sites/default/files/2020-08/Checklist_for_Analytical_Cross_Sectional_Studies.pdf.

[20] Critical Appraisal Skills Programme (2018). CASP Qualitative Checklist. https://casp-uk.net/casp-tools-checklists/qualitative-studies-checklist/.

[21] Brooks F (1998). Women in general practice: responding to the sexual division of labour?. Soc Sci Med.

[22] French F, Andrew J, Awramenko M (2006). Why do work patterns differ between men and women GPs?. J Health Organ Manag.

[23] Gravelle H, Hole AR, Santos R (2011). Measuring and testing for gender discrimination in physician pay: English family doctors. J Health Econ.

[24] Baker M, Williams J, Petchey R (1995). GPs in principle but not in practice: a study of vocationally trained doctors not currently working as principals. BMJ.

[25] Johnson N, Hasler J, Hayden J (1998). The career outcomes for doctors completing general practice vocational training 1990–1995. Br J Gen Pract.

[26] Johnson N, Hasler J, Mant D (1993). General practice careers: changing experience of men and women vocational trainees between 1974 and 1989. Br J Gen Pract.

[27] Newman P (2011). Releasing potential: women doctors and clinical leadership. https://s3-eu-west-2.amazonaws.com/images.pulsetoday.co.uk/wp-media-folder-pulse-today/wp-content/uploads/c_files/uploads/2012/10/03/o/n/m/1332847620_DgNz_releasing_potential_women_doctors_and_clinical_lea-2.pdf.

[28] Osler K (1991). Employment experiences of vocationally trained doctors. BMJ.

[29] Watts CE (2018). The feminisation of the medical profession in England: implications and responses [PhD Thesis].

[30] Wedderburn C, Scallan S, Whittle C, Curtis A (2013). The views and experiences of female GPs on professional practice and career support. Educ Prim Care.

[31] Young G (1983). Newcastle vocational trainees 1976-80: are they doing the work they wanted? Newcastle branch of women in medicine. Br Med J (Clin Res Ed).

[32] French F, Andrew J, Awramenko M (2005). General practitioner non-principals benefit from flexible working. J Health Organ Manag.

[33] Lawrence B (1987). Gender and general practice: the single-handed women general practitioner [PhD Thesis].

[34] Leese B, Young R, Sibbald B (2002). GP principals leaving practice in the UK: similarities and differences between men and women at different career stages. Eur J Gen Pract.

[35] Pinder R (1998). On the margins: belonging in general practice for women part-timers and non-principals. Fam Pract.

[36] Warren VJ, Wakeford RE (1989). ’We’d like to have a family’—young women doctors’ opinions of maternity leave and part-time training. J R Soc Med.

[37] Morris S, Goudie R, Sutton M (2011). Determinants of general practitioners’ wages in England. Health Econ.

[38] Wordsworth S, Skåtun D, Scott A, French F (2004). Preferences for general practice jobs: a survey of principals and sessional GPs. Br J Gen Pract.

[39] Young R, Leese B, Sibbald B (2001). Imbalances in the GP labour market in the UK: evidence from a postal survey and interviews with GP leavers. Work Employ Soc.

[40] House of Commons Health Committee (2004). GP out-of-hours services: Fifth report of session 2003–04. https://publications.parliament.uk/pa/cm200304/cmselect/cmhealth/697/697.pdf.

[41] Jefferson L, Golder S, Essex H (2023). Exploring gender differences in uptake of GP partnership roles: a qualitative mixed-methods study. Br J Gen Pract.

[42] Jefferson L, Golder S, Heathcote C (2022). GP wellbeing during the COVID-19 pandemic: a systematic review. Br J Gen Pract.

[43] National Institute for Health and Care Research (2023). Statement of intent: Integrating sex and gender into health and care research. https://www.nihr.ac.uk/integrating-sex-and-gender-health-and-care-research.

[44] Dacre J, Shepherd S (2010). Women and medicine. Clin Med (Lond).

[45] Hutchinson J, Gibson J, Kontopantelis E (2024). Trends in full-time working in general practice: a repeated cross-sectional study. Br J Gen Pract.

[46] British Medical Association (BMA) (2022). Salaried GPs handbook. https://www.bma.org.uk/media/6582/salaried-gp-handbook-updateoct2022.pdf.

[47] Bevan S, Bajorek Z, Edwards M, Plowden Roberts C (2022). Job crafting and flexible working in general practice (Institute for Employment Studies). https://www.employment-studies.co.uk/resource/job-crafting-and-flexible-working-general-practice.

[48] NHS England (2020). Investment and evolution: update to the GP contract agreement 2020/21– 2023/24. https://www.england.nhs.uk/publication/investment-and-evolution-update-to-the-gp-contract-agreement-20-21-23-24/.

[49] Dale J, Potter R, Owen K (2015). Retaining the general practitioner workforce in England: what matters to GPs? A cross-sectional study. BMC Fam Pract.

[50] Kelly M, Berlin A, Abrams R, Park S (2019). Pitfalls and pleasures of pick-and-mix careers: portfolio working and whole-person medicine in general practice. Br J Gen Pract.

[51] Khan N (2024). A failure to retain GP retention schemes. Br J Gen Pract.

[52] Palmer WL, Rolewicz L, Tzortziou Brown V, Russo G (2025). A hole in the bucket? Exploring England’s retention rates of recently qualified GPs. Hum Resour Health.

